# Exploring the Photocatalytic Cleavage Pathway of the β‐5 Linkage Lignin Model Compound on Carbon Nitride

**DOI:** 10.1002/cssc.202400955

**Published:** 2024-11-07

**Authors:** Junhong Liu, Kathryn Ralphs, Christopher W. J. Murnaghan, Nathan Skillen, Gary N. Sheldrake, Philip McCarron, Peter K. J. Robertson

**Affiliations:** ^1^ School of Chemistry and Chemical Engineering Queen's University Belfast David Keir Building, Stranmillis Road Belfast BT9 5AG United Kingdom of Great Britain and Northern Ireland; ^2^ Institute for Global Food Security School of Biological Sciences Queen's University Belfast Chlorine Gardens Belfast BT9 5DL United Kingdom of Great Britain and Northern Ireland

**Keywords:** Photocatalysis, Biomass, Lignin model compound, Carbon nitride, Valorization

## Abstract

As a globally abundant source of biomass, lignocellulosic biomass has been the centre of attention as a potential resource for green energy generation and value‐added chemical production. A key component of lignocellulosic biomass, lignin, which is comprised of aromatic monomers, is a potential feedstock for value added chemical production. The cleavage processes of the linkages between monomers to obtain high value products, however, requires significant investigation as it is a complex, non‐facile process. This study focuses on the photocatalytic valorization of a β‐5 lignin model compound, a key linkage in the lignin structure. It was found that greater yields of aromatic products were obtained from the photocatalytic conversion of β‐5 lignin model compound using carbon nitride (CN) when compared to Evonik P25 titanium dioxide (TiO_2_). Products of the β‐5 model compound photocatalytic conversion were determined and C−C bond cleavage was observed. It was also determined that the solvent participated in the reactions with the introduction of a cyano group to one of the products. Radical quenching experiments revealed that superoxide radicals participated in the CN photocatalytic conversion. These results reveal for the first time the products and possible mechanism of the photocatalytic transformation of β‐5 model compounds using CN photocatalysis.

## Graphical abstract

Photocatalytic conversion of biomass is becoming increasingly important, with the photocatalytic transformation of lignin being both attractive and challenging. This article presents the first use of graphitic carbon nitride for the photocatalytic conversion of a β‐5 model compound, revealing depolymerization products.

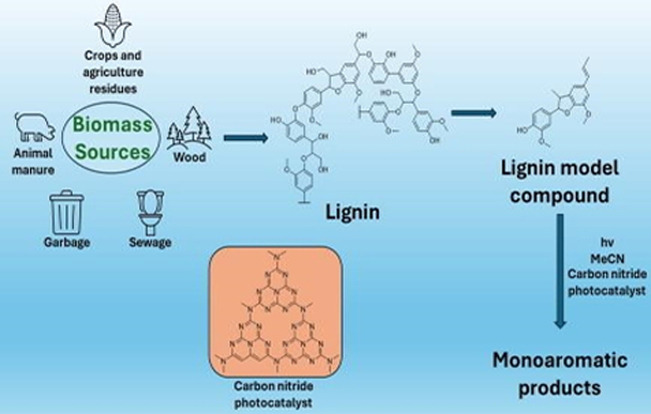

## Introduction

With increasing CO_2_ emissions each year, it is imperative that there is a transition from fossil fuels to meet Net Zero targets,^[1]^ which have been set to ensure the rise in global average temperature does not exceed 2 °C above pre‐industrial levels as agreed in the 2015 Paris agreement.^[2]^ For example, in 2019 the UK was one of the first countries to enshrine into law the objective of ensuring greenhouse gas emissions would be net zero, compared to 1990 levels, by 2050.^[3]^

With abundant renewable biomass sources, bioenergy could provide sustainable and low‐carbon energy which supports the Net Zero energy transition.^[4]^ Bioenergy is already an important part of the energy economy. In 2024 biomass supplied (on average) 6.3 % of the UKs electricity generation, which equates to approx. 1.9 GW. Overall, in 2024, renewables (including biomass) provided 44.4 % of electricity to the grid, which is just over 13 GW of production.[^5^, ^6^] To meet the carbon emission reduction targets, the proportion of modern bioenergy which is considered as *“biomass used alongside modern heating technologies, power generation and transport fuels as opposed to traditional wood‐burning methods commonly used for heating and cooking in developing countries*”^[7]^ needs to increase. Biomass conversion technology is also one of the few systems that could offer ‘negative emissions’ if it is coupled with Carbon Capture Utilization and Storage (CCUS) processes. Based on hydrogen production, wood gasification with CCS could generate ~‐150 g CO_2_ e/MJ H_2_ (LHV). By comparison, fossil fuel SMR produces ~90 g CO_2_ e/MJ H_2_ (LHV).^[8]^ Consequently, research into converting biomass into fuels and high‐value chemicals has received a significant amount of attention in recent years.[^9^, ^10^, ^11^, ^12^, ^13^, ^14^, ^15^, ^16^]

Over the past decade there has been a substantial level of research interest in the application of photocatalytic reforming of lignocellulosic biomass.[^17^, ^18^, ^19^, ^20^, ^21^] This abundant sustainable feedstock is a promising substrate for clean energy production with cellulose and hemicellulose having been widely investigated and demonstrated to be potential substrates for the generation of hydrogen and value‐added chemicals.[^22^, ^23^] Using sunlight as the energy source under ambient temperature and pressure, photocatalytic reforming of biomass to produce hydrogen or high‐value chemicals is recognized as a potential low‐carbon and more sustainable technology^[24]^ compared to conventional thermochemical methods.^[25]^ Lignin, a complex and abundant biopolymer found in plant cell walls, is one of the most promising renewable resources as the largest natural large scale source of aromatics.^[12]^ Historically considered a by‐product of the pulp and paper industry, lignin has gained increasing attention in recent years due to its potential as a valuable feedstock for the production of high‐value chemicals and materials.^[26]^ Lignin can be depolymerized to produce various phenolic compounds, such as vanillin, syringaldehyde, guaiacol, and catechol.^[27]^ These compounds have applications in the manufacturing of food flavorings, pharmaceuticals and fragrances, or as chemical intermediates for the synthesis of polymers and resins.^[28]^

**Figure 1:**
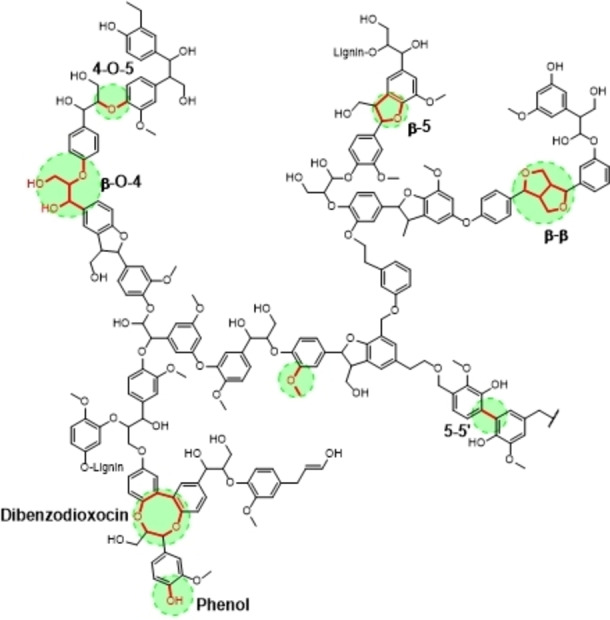
**S**chematic showing linkages present between lignin monomers.

The reforming of lignin, however, remains challenging due to its complicated three‐dimensional amorphous polymer structure which consists of three aromatic main units: syringyl, guaiacyl, and p‐hydroxyphenyl units.[^29^, ^30^] The monomers of lignin are linked mainly by ether or carbon‐carbon bonds, with more than two thirds ether bonds and the rest carbon‐carbon bonds. The linkages of the lignin monomers are mainly β‐O‐4 (40–60 %), β‐5 (4–12 %), and 5–5 (4–25 %). There are also small amounts of α‐O‐4 (4–8 %), β‐β (2–7 %), 4‐O‐5 (4–7 %), β‐1 (3–7 %) and α‐O‐γ, etc.[^31^, ^32^] (Figure 1). β‐O‐4 linkages being the most abundant linkage found in native lignin has been the first and most extensively studied[^33^, ^34^, ^35^, ^36^, ^37^, ^38^] as model compounds, while the other linkages have not yet been investigated in as much detail.[^39^, ^40^, ^41^]

Our group has previously reported the photocatalytic degradation of β‐5 linkages using a TiO_2_ photocatalyst activated with UV‐light.^[42]^ Under low power UV‐light emitting diode (LED) irradiation at 370 nm, complete conversion (to the limit of detection) of the β‐5 compounds (6.3×10^−3^ mg ml^−1^ min^−1^) was achieved together with the formation of a number of reaction intermediates. While a number of intermediates in the TiO_2_‐catalysed degradation of β‐5‐linked model compounds were identified, guaiacol or guaiacol‐based products were not detected at the end of the reaction period.^[42]^ This may have been due to non‐selective oxidation as a result of the strong oxidising capacity of the OH radical (2.8 eV vs NHE) generated on the photocatalyst surface. Therefore, there is a need for a photocatalyst that can selectively convert β‐5 model compounds under visible light to obtain the targeted high‐value monomers. In other studies, the selective conversion of lignin model compounds on graphite like carbon nitride (CN) and derived materials has been demonstrated. In previous studies it was confirmed that modified CN can selectively convert β‐O‐4 and β‐1 lignin model compounds.[^37^, ^43^] Consequently, in this work CN,[^44^, ^45^, ^46^, ^47^] a common non‐metallic semiconductor photocatalytic material, has been used for the photocatalytic conversion of the β‐5 model compound. CN, which consists of carbon and nitrogen elements in a layered graphite‐like structure, is stable, and the threat of secondary pollution to the environment is lower than that with nano‐TiO_2_ materials.[^48^, ^49^] CN has a lower bandgap (Eg ≈2.8 eV) and conduction band position than TiO_2_ (Eg ≈3.2 eV), and as a result is not believed to generate hydroxyl radicals directly from water. Consequently, this should inhibit degradation of monomeric products. An additional key advantage of CN photocatalysts is their ability to absorb visible light, which constitutes a significant portion of the solar spectrum. Moreover, CN has been used by other researchers to verify the feasibility of photocatalytic cleavage of β‐O‐4 model compounds and to demonstrate that CN can selectively break these ether linkages.[^37^, ^50^] In light of these results with the β‐O‐4 model compounds, CN was synthesized and investigated for the cleavage of the β‐5 linkage and compared to TiO_2_ with identification of monomeric products. This paper details an investigation of the performance, potential monomer products and cleavage mechanism for degradation of β‐5 lignin model compound using a CN photocatalyst under visible light irradiation.

## Results and Discussion

### Characterisation of the CN Photocatalyst

**Figure 2:**
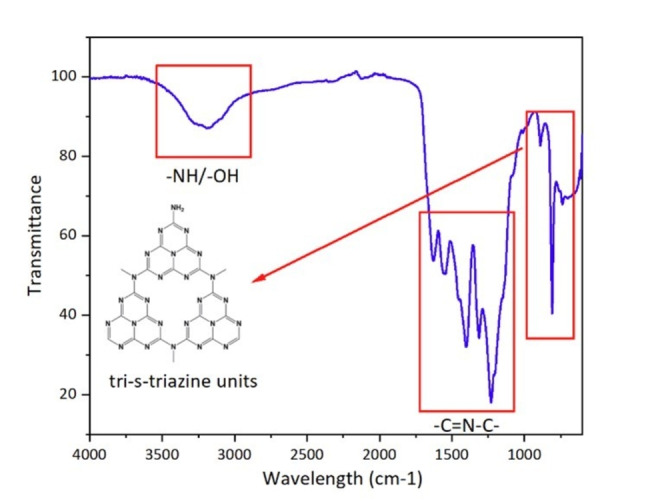
FT‐IR spectrum of CN.

**Figure 3:**
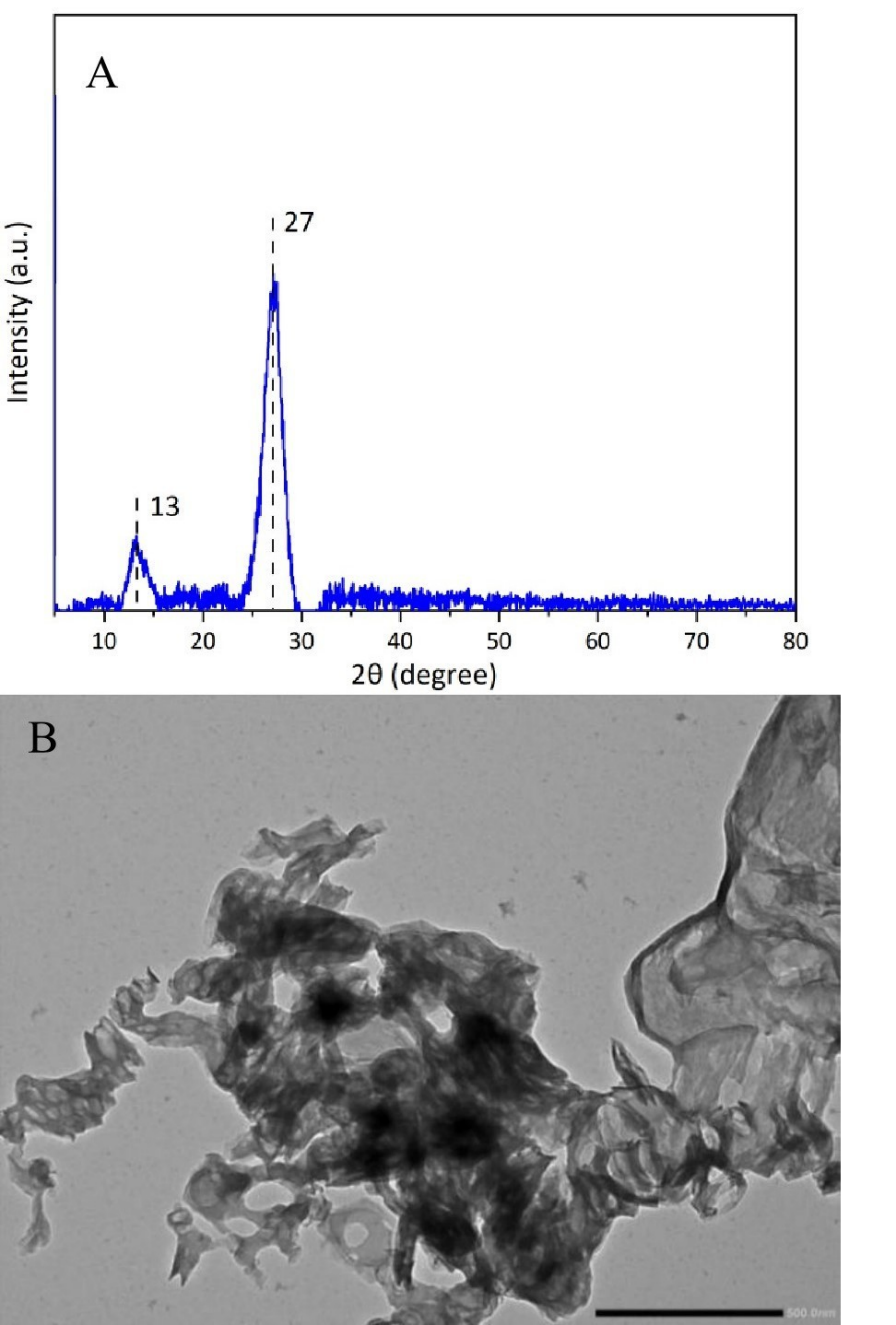
**A)** XRD pattern and B) TEM images of prepared CN.

The CN photocatalyst was initially characterized using X‐ray diffraction (XRD), Fourier transform infra‐red spectroscopy (FTIR), Brunauer‐Emmett‐Telle surface area (BET) analysis, transmission electron microscopy (TEM) and scanning electron microscopy (SEM). As shown in Figure 2, a series of characteristic peaks for CN were observed in the FT‐IR spectrum.^[51]^ A series of absorption bands from 1200–1600 cm^−1^ which correspond to the stretching vibration modes of C−N(−C) −C or bridging C−NH−C units were observed. The wide band between 3000–3500 cm^−1^ is attributed to the N−H vibration. There was a sharp peak located at 808 cm^−1^ in the fingerprint region that has been attributed to the breathing mode of tri‐s‐triazine units^[52]^. The XRD spectrum and TEM images for the CN photocatalyst are displayed in Figure 3. In the XRD diffractogram the two peaks located at 13° and 27° represent the interlayer long range order and stacking of carbon and nitrogen under van der Waals forces.^[53]^ TEM analysis confirmed that the CN material consists of multiple layers stacked on top of one another. Based on the above results, it is clear that the synthesized material has a layered graphite‐like structure. BET analysis (Table 1) revealed a surface area of 48.92 m^2^ g^−1^ which was comparable to the surface area (48.2 m^2^ g^−1^) reported previously for this material.^[37]^

**Table 1:** BET surface area and pore volume of CN.

Sample	Surface area (m^2^/g)	Pore volume (cm^3^/g)	Pore size (Å)
CN	48.92	0.13	82.50
C3N4‐U^[33]^	48.20	0.15	29.00

**Figure 4:**
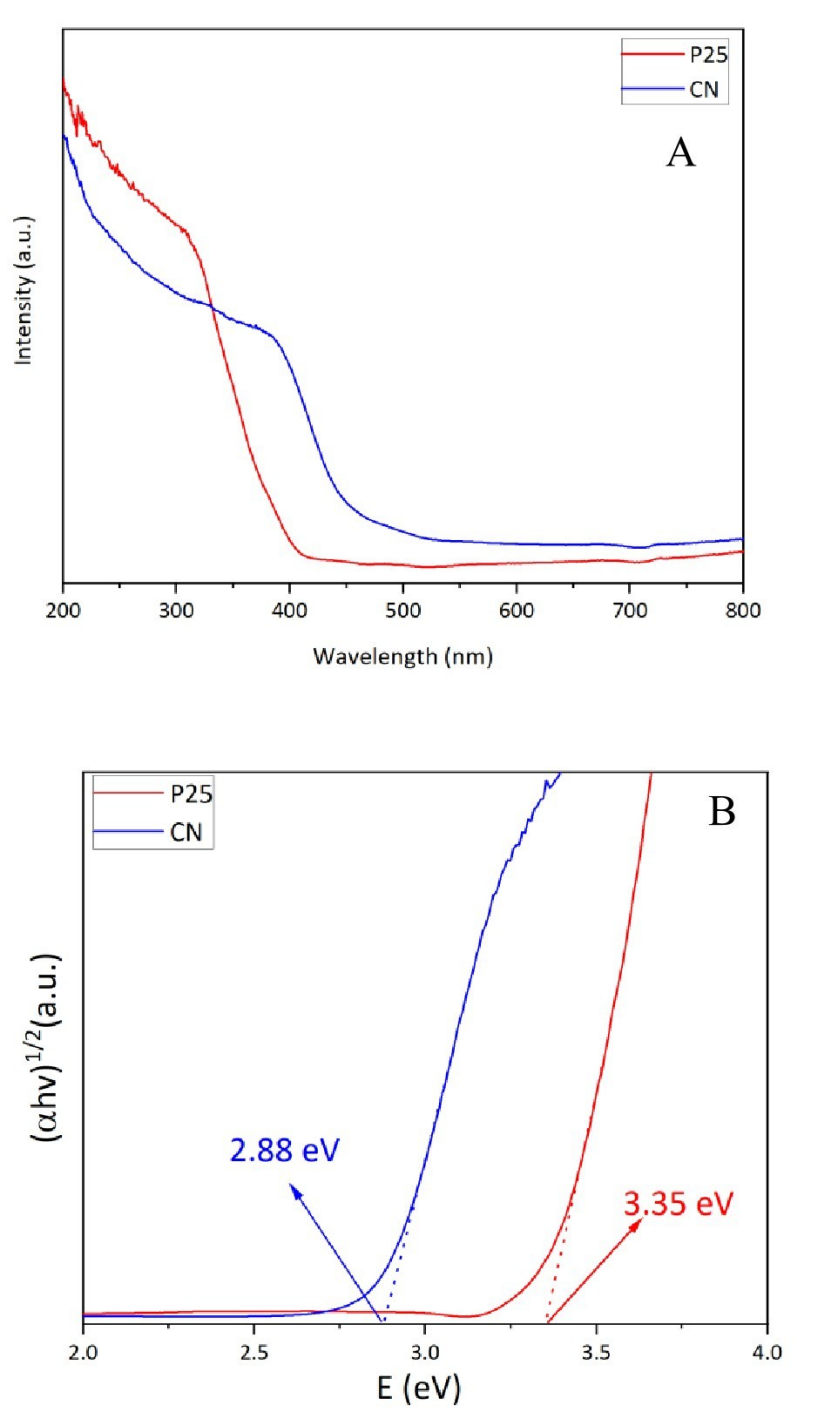
**A)** UV‐vis DRS spectra and B) plots of (αhυ)1/2 versus photon energy of CN and P25.

The CN bandgap width was determined using UV‐visible diffuse reflectance spectroscopy (UV‐vis DRS). In Figure 4 the UV‐vis DRS spectra of the CN and TiO_2_ P25 photocatalyst materials shows the adsorption wavelength edges located at 451 nm and 391 nm respectively. This spectrum implies that the prepared CN is photo‐responsive in the visible light region of 400–450 nm whereas P25 TiO_2_ is not. Estimated from the tangent intercept of the (αhν)1/2 vs photon energy curve, the band gap energy (Eg) of CN is 2.88 eV which is consistent with a previous report.^[37]^

### Comparison of Photocatalytic Performance of CN and P25 TiO_2_ Photocatalysts

**Figure 5:**
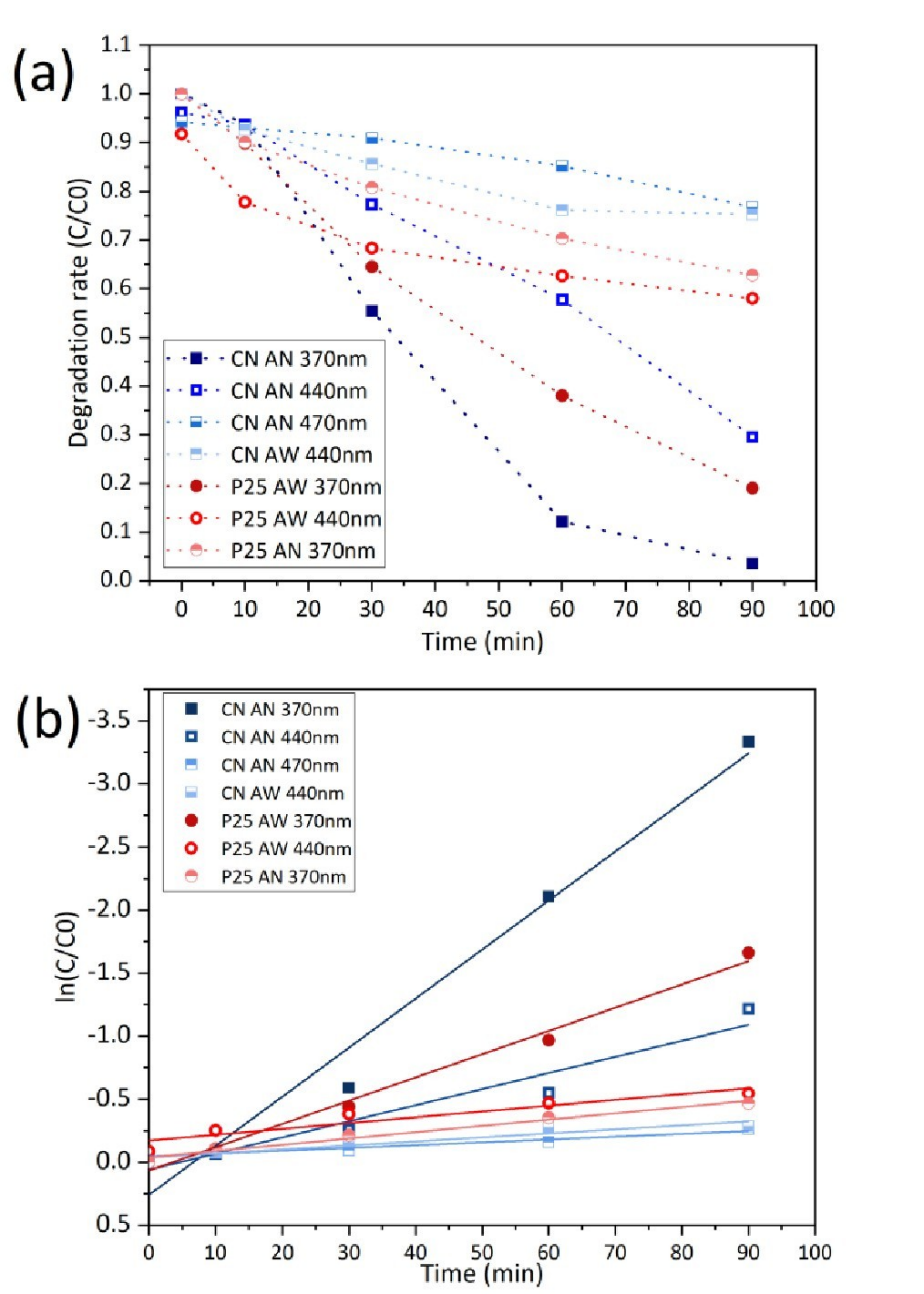
β‐5 photocatalytic degradation with CN and P25 TiO_2_ under different light wavelengths and solvents(a) and plot ln(C/C0) verse time(b). (AN: pure acetonitrile, AW: acetonitrile/water mixture (50/50 v/v)).

To investigate the performance of the prepared CN photocatalyst for the degradation of the β‐5 lignin model, photocatalytic degradation experiments were carried out with both this material and an Evonik P25 TiO_2_ photocatalyst. For comparison with previous work, and to investigate the performance under visible light in this experiment, 370 nm, 440 nm and 470 nm LEDs were used as irradiation sources. As shown in Figure 5, in an acetonitrile solution, 96.4 % of β‐5 was degraded by the CN material within 90 min under 370 nm irradiation. In comparison while using a P25 TiO_2_ photocatalyst, only 37.2 % degradation was achieved under the same reaction conditions. When the irradiation source was changed to visible light (440 nm), CN still demonstrated a good level of degradation, with 66.6 % of β‐5 degraded over the same reaction time period. When a 470 nm LED was utilized as the illumination source, the degradation rate decreased rapidly, with only 17.5 % degradation following 90 min of irradiation. Interestingly, however, in an acetonitrile/water solution as used in our previous work, CN′s degradation capacity was greatly inhibited. Only 24.8 % degradation was obtained over 90 min under irradiation with the 440 nm illumination. The P25 material was able to achieve a 42.0 % degradation using the mixed acetonitrile/water solvent under identical experimental conditions. This indicates that CN has demonstrated photocatalytic activity for β‐5 degradation in the UV−A and near UV‐visible regions. The bandgap of CN is narrower than that of P25, allowing it to obtain a wider light absorption region than that of P25, but at the same time reducing the redox capacity of CN. Therefore, this may lead to different reaction mechanisms for CN and P25. In order to obtain a reasonable level of degradation, it was necessary to use only acetonitrile as a solvent.

**Figure 6:**
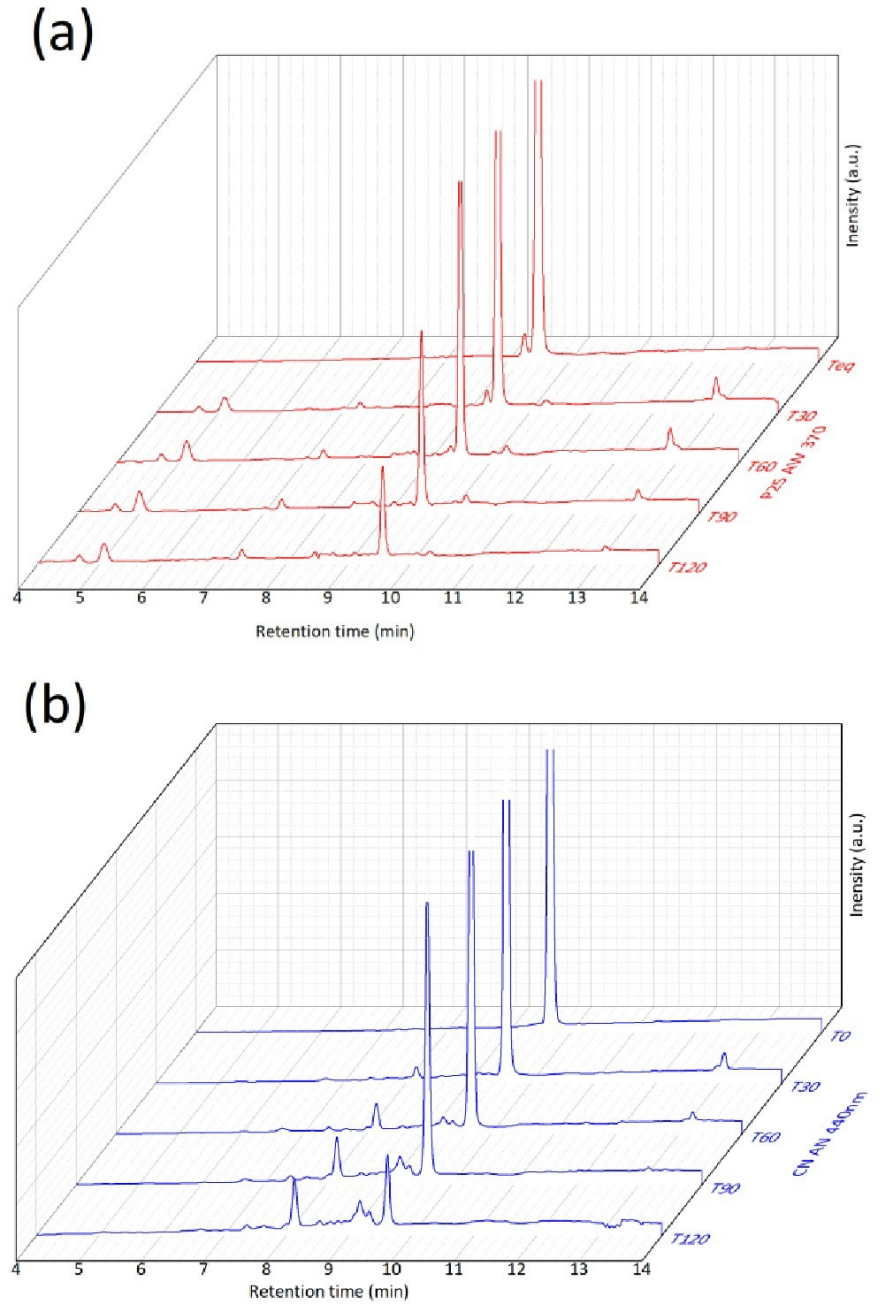
Stack HPLC chromatograms of extracted substrate across reaction time periods with P25 (a) and CN (b).

In addition to investigating the performance of CN for the degradation of β‐5, it was important to examine the by‐products generated as part of this degradation process. The HPLC chromatograms shown in Figure 6 are taken from substrate analysis at different time periods under CN and P25 photocatalytic degradation reactions. The peak located at around 9.8 min (R_T_ 9.8) is characteristic of the β‐5 substrate. During 0–30 min, P25 TiO_2_ and CN simultaneously produced products that peaked at 13 min (R_T_ 13). After 30 min as the intensity of the R_T_ 13 peak decreased, the product peaks R_T_ 5.1 and R_T_ 7.3 were produced under P25 TiO_2_ photocatalysis, whereas the product peaks R_T_ 8.1, 9.1 and 9.3 were produced under photocatalysis using the CN material. This implies that P25 TiO_2_ and CN appeared to follow the same reaction pathway in the first 30 min of photocatalysis. After 30 min, however, the two photocatalysts followed different pathways for the β‐5 substrate resulting in different degradation products.

### Investigation of Photocatalytic Conversion Mechanism of β‐5 Model Compound on CN

**Figure 7:**
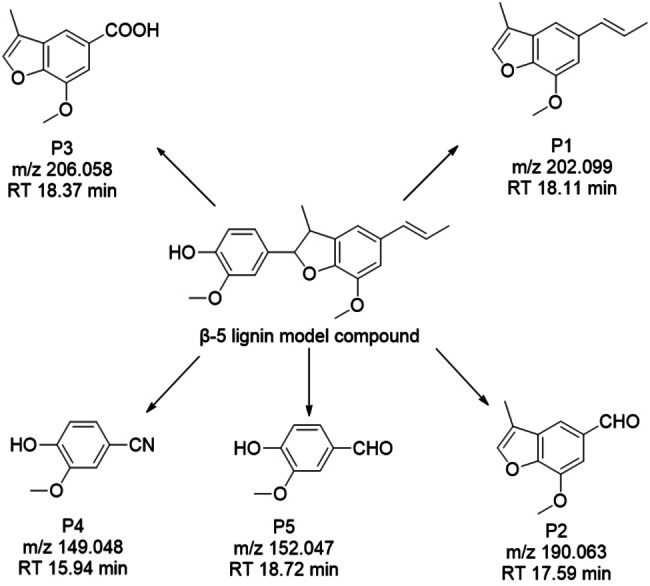
Potential products from β‐5 conversion on CN analyzed by GC‐MS and detailed structure of β‐5 substrate.

For a better understanding of the products generated and the mechanism of CN photocatalytic conversion of the β‐5 model compound, GC‐MS was utilized for the analysis of reaction solutions from different reaction times and irradiations. From the results shown in Figure S1–S5 and, Table S1 and S2 in the supplementary information file, it can be noted that the products obtained from the photocatalytic conversion of β‐5 by CN under different peak wavelengths (e. g.) are consistent. In order to compare with P25 TiO_2_ under the same conditions, the reaction solution after 1 and 4 under 370 nm irradiation were used for analysis. Based on previous studies, the identified products are shown in Figure 7. Notably, a guaiacol‐based monomeric compound which was not observed in previous work was detected as a product of the CN photocatalytic process, labelled as products P4 and P5 (Figure 7). Products **P1**, **P2** and **P3** are proposed to have been produced by cleavage of the α−C−C bond (labelled on β‐5 dimer in Figure 7) between the benzofuran and the aromatic ring, with such an elimination being proposed to result in the formation of an alkene in the furan ring. The above postulated products are consistent with the previous work, where the α−C−C bond of the model compound was cleaved. Furthermore, product **P2** is proposed to undergo oxidation of the propenyl sidechain to furnish an aldehyde, while P3 is further oxidised to a carboxylic acid. Unusually, however, product P2 was not further degraded but remained stable after cleavage under CN photocatalysis, as it was detected in both the 4 h reaction samples, with the signal intensity remaining stable with only about 6.5 % loss. These findings suggest that P2 has not undergone oxidation to the carboxylic acid within the reaction solution and that P3 was formed by a potentially different mechanism. Vanillin (**P5**) was identified by HPLC and confirmed by GC‐MS (Figure S3 and S4). P5 is thought to be produced via cleavage of side‐chain olefin and subsequent bond cleavages within the benzofuran ring.

**Figure 8:**
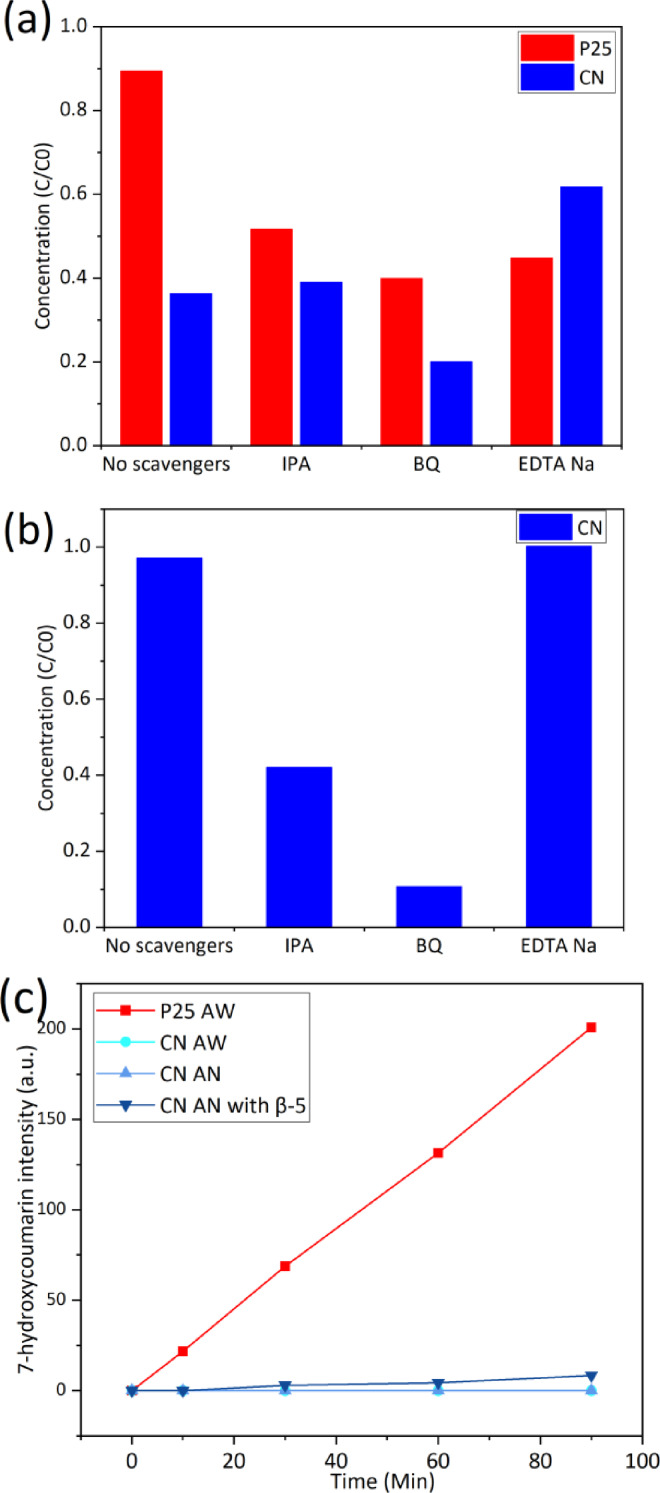
Results of radical quenching experiments for P25 and CN (a) in acetonitrile/water (v/v 50 : 50) and acetonitrile (b). Hydroxyl radical yield comparison between P25 and CN in acetonitrile/water (v/v 50 : 50) and CN in acetonitrile with and without β‐5 (c).

In order to gain a deeper understanding of the mechanism of photocatalytic conversion of β‐5 model compounds by the CN photocatalyst, free radical experiments were carried out to elucidate which Reactive Oxygen Species (ROS) were generated as part of the photocatalytic process. Isopropanol (IPA), p‐benzoquinone (BQ) and ammonium oxalate (AO)/EDTA Na were utilized as hydroxyl radical (**⋅**OH), superoxide radical (**⋅**O_2_^−^) and photogenerated holes (h^+^) quenchers, respectively, and the results are shown in Figure 8(a) and (b). It is noteworthy that the reaction was significantly inhibited after the addition of both IPA and BQ. According to other reports, IPA is photocatalytically oxidised to acetone by CN photocatalysts^[53]^, hence it is likely that IPA is the preferred substrate leading to the inhibition of the β‐5 conversion reaction. To exclude the interference of IPA and to verify whether ⋅OH was generated and involved in the reaction, coumarin was used as a hydroxyl radical probe in this study.[^54^, ^55^]

To detect the production of **⋅**OH radicals, coumarin was used as a probe. Coumarin can trap **⋅**OH and produce hydroxycoumarin compounds with 7‐hydroxycoumarin fluorescing at 405 nm, and the presence and intensity of fluorescence can be used to determine the generation and the concentration of **⋅**OH radicals, as shown in Figure 8(c). In mixed acetonitrile/water solutions no 7‐hydroxycoumarin was generated by the CN photocatalyst in acetonitrile and acetonitrile/water solutions in the absence of β‐5. Interestingly, 7‐hydroxycoumarin was, however, detected when β‐5 was added to the reactor. Since CN cannot produce ⋅OH radicals in pure acetonitrile, 7‐hydroxycoumarin could only result from hydrogen peroxide produced by the reduction of oxygen by CN, which subsequently produces hydroxyl radicals. Moreover, in the process of generating hydrogen peroxide, protons were likely supplied by β‐5. These results would suggest that ⋅O_2_^−^ are the dominant ROS in the photocatalytic degradation reaction of β‐5 using a CN photocatalyst.
(1)

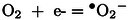



(2)

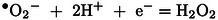




Additionally, it was observed that the dosage of the photogenerated hole scavenger (ammonium oxalate and EDTA Na) accelerated the photocatalytic degradation of β‐5 both in pure acetonitrile and in the mixed acetonitrile/water solution. The fact that the reaction was accelerated by the depletion of holes suggests that the hole scavenger promoted the separation of photogenerated hole‐electron pairs, thus enhancing the generation of ⋅O_2_^−^. Considered alongside the results of other radical quenching experiments, superoxide radicals appear to be the predominant ROS involved in the CN photocatalytic degradation reaction of β‐5.

**Figure 9:**
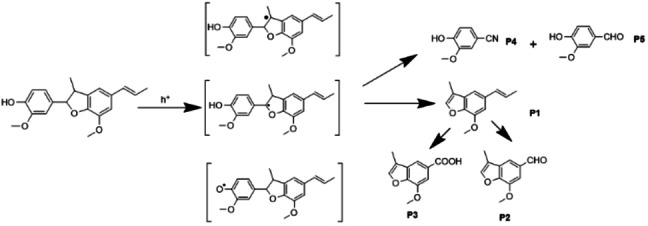
Schematic of proposed β‐5 conversion mechanism on CN.

Combining all the above results, a schematic of the mechanism of the photocatalytic degradation of the β‐5 substrate is proposed in Figure 9. It is proposed that following light excitation of the CN photocatalyst, charge separation occurs in the CN catalyst then photogenerated electrons and holes are generated on the surface. Photogenerated electrons on the CN surface subsequently reduce oxygen into a **⋅**O_2_^−^. Meanwhile, photo‐generated holes extract protons from β‐5, ultimately combining with **⋅**O_2_^−^ to generate hydrogen peroxide. Product **P2** was derived from **P1** and accounts for the cleavage in side‐chain olefin. According to Zhang et. al.,^[56]^ The double bond of the olefin is cleaved to form an aldehyde with CN, under blue LED light conditions with acetonitrile as the solvent, as a result of direct hole oxidation and reaction via superoxide radical anions generated via the valence band reduction of oxygen. Therefore, the side‐chain olefin **P1** is oxidized into **P2**. The aldehyde group is subsequently further oxidized to a carboxyl group, resulting in product **P3**. As for **P5**, the introduction of the aldehyde group may be generated by the involvement of superoxide radicals in the reaction.

Additionally, acetonitrile not only functions as a solvent but is also proposed to participate in the transformation reaction of β‐5. The cyano group introduced in product **P4** is likely derived from the solvent acetonitrile. According to Addamo et al.,^[57]^ the reaction of **⋅**OH with acetonitrile has the ability to produce cyano radicals. It was however not possible to confirm the presence of cyano radicals in this study, but this will be investigated later using isotopically labelled acetonitrile solvents. In the absence of such a protocol for cyano radical detection, it is still our belief that the generation of product **P4** arises from the solvent participating in the reaction. With this in mind, it is thought that CH_3_CN is likely to also have a significant impact on the degradation rate and pathways of product formation

## Conclusions

In the present study, building on the previous research on the photocatalytic degradation^[36]^ of β‐5 lignin model compounds by TiO_2_, a typical non‐metallic semiconductor CN was successfully synthesised and used as a photocatalyst for the degradation of this material. The results of this work demonstrated that CN effectively degraded the β‐5 model compounds under both UV and visible light irradiation, while generating different decomposition by‐products compared to those produced when a P25 TiO_2_ material was used as the photocatalyst. The reaction solution was analyzed by HPLC, and the products identified by GC‐MS with the results showing that the possible initial fracture site was between the α‐C−C bond between the benzofuran and the aromatic ring. Furthermore, the ROS involved in the photocatalytic reaction were also investigated. Using the CN photocatalyst the degradation of β‐5 was believed to result from reaction with **⋅**O_2_^−^ as indicated from radical quenching studies. The role and the mechanism of how acetonitrile participated in the reaction, however, requires further investigation. This reaction demonstrates that CN photocatalysts may be highly selective materials for the conversion of β‐5 compounds into monomeric high‐value compounds.

## Experimental

### Materials

All reagents were used as received without further treatment. Titanium dioxide (P25) was purchased from Evonik. Isopropanol, benzoquinone, ammonium oxalate, acetonitrile, isoeugenol, iron (III) chloride, ethanol, dichloromethane (DCM) and coumarin were all purchased from Sigma‐Aldrich. Deionized water was obtained from an in‐house distillation system and used without further treatment.

### Preparation of CN Photocatalyst Materials

CN was synthesized by thermal condensation method.^[50]^ 10 g urea was placed in a crucible with lid and placed in furnace at 550 °C for 2 h. The material was allowed to cool to room temperature and then the obtained yellow powder was flushed with deionized water 50 mL for 3 times and collected by centrifugation each time. Finally, pale‐yellow CN was obtained after drying in an oven for 24 h at 65 °C.

### Preparation of β‐5 Lignin Model Compound

β‐5 lignin model compound was prepared by following a method reported in previous study.^[36]^ A reaction flask was charged with isoeugenol (5.0 g, 30 mmol) and made into solution by addition of ethanol and water (25 mL and 57 mL respectively) and allowed to stir for 2 minutes to ensure adequate dispersion of substrate. Iron (III) chloride (2.96 g, 18.2 mmol, 0.6 eq) was added to this solution and the flask was then stoppered and shaken vigorously for 1 minute providing a blue‐black solution which was then stored in the freezer for 3 days. After this period of time, the crystals formed were separated from the solution by suction filtration, the separated crystals were then washed with ice‐cold ethanol (20 mL). The washed crystals were then suspended in the minimum amount of DCM and then passed through a short silica plug. The plug was then washed with DCM (100 mL) and the filtrate stripped down to provide the product as a white solid in 0.94 g, 19 % yield. The NMR spectra and UV‐vis spectra are available in supporting information.

### Photocatalysis Procedure

The photocatalytic experiments were all carried out in cylindrical glass vials as shown in Figure. S7. The cylindrical reactor had a diameter of 500 mm and was surrounded by an LED array which was mounted onto a 680 mm diameter stainless steel support. The array was comprised of a LED strip which consisted of LEDs with a peak wavelength of 370 or 440 nm. The LED arrays were operated with a V_F_ =12 dcV and I^F^ =0.3 A.

In order to accurately monitor the output of the LED strip, the photon flux was measured using the potassium ferrioxalate actinometry method.^[58]^ The photon flux was calculated using Equation 3:
(3)

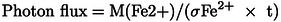




Where M(Fe^2+^) (mol) was determined by the potassium ferrioxalate method, σFe^2+^ was set at 0.97 and t was the time (min) the actinometry solution was irradiated for. From the experimentally obtained data, it can be calculated that the photon flux inside the reactor under the irradiation of 370 nm and 440 nm LED cages is 1.33×10^−6^ mol/min and 2.28×10^−6^ mol/min, respectively.

The β‐5 model compound (12.5 mg, giving a concentration of 250 mg/L) and the catalyst (25 mg) were added to 50 mL of either acetonitrile or acetonitrile/water (50/50 v/v) in the glass reactor. The mixture was stirred continuously by a magnetic stirrer at a rate of 400 rpm. The experiment was initially stirred for 30 min under dark conditions to reach an adsorption equilibrium. The light source was then switched on and the voltage was set to 12 V running at 6 W power. Samples were taken at set times and filtered through a 0.2 μm nylon syringe filter.

Reactive oxygen species quenching experiments were conducted under the same conditions and procedure as the photocatalytic experiment except adding in isopropanol (IPA), p‐benzoquinone (BQ) and ammonium oxalate (AO)/EDTA Na as hydroxyl radical (⋅OH), superoxide radical (⋅O_2_^−^) and photogenerated holes (h^+^) quenchers, respectively. All quenchers were dosed with a concentration of 0.05 mmol/L.

Hydroxyl radical probe experiments were conducted under the same conditions as the photocatalytic experiment except reaction substrates were replaced by 50 mL 100 μM coumarin acetonitrile solution or acetonitrile/water solution or acetonitrile with 250 mg/L β‐5 compound solution.

The above reaction procedure was carried out at room temperature. The temperature was continuously monitored by a Pico Log type K thermocouple and the results are shown in Figure S8. The temperature increased from 21 °C to a maximum of 25 °C during the reaction. The temperature difference throughout the reaction was approximately 4 °C, so the effect of temperature on the reaction was deemed to be negligible.

### Analytical Methods

HPLC–UV analysis for the photocatalytic performance experiment samples were performed using an Agilent 1100 HPLC system equipped with a photodiode array UV‐Visible detector with a Phenomenex Kinetex® C18 analytical column (150 mm×4.6 mm, 100 Å). Two mobile phases were used: (A) 0.2 % aqueous acetic acid and (B) methanol over the following gradient: 50 : 50 (A : B) to 100 % B (0–6 min) and then back to 50 : 50 (A : B) (6–10 min). The flow rate was 1 mL min^−1^ and the injection volume was 5 μL. Under this condition β‐5 compound eluted at 5.9 min.

Analysis of samples at different reaction times were performed using the same system but with a Phenomenex Kinetex® Phenyl‐Hexyl analytical column (150 mm×4.6 mm, 100 Å). Two mobile phases were used: (A) 0.2 % aqueous acetic acid and (B) methanol over the following gradient: 50 : 50 (A : B) to 100 % B (0–10 min) and then keep it until 15 min. The flow rate was 1 mL min^−1^ and the injection volume was 8 μL.

Analysis of samples from hydroxyl radical probe experiments were performed with same system and column above.^[59]^ Two mobile phases were used: (A) 89 % water/10 % methanol/ 1 % acetic acid and (B) 89 % methanol/10 % water/1 % acetic acid over the following gradient: 90 : 10 (A : B) to 30 : 70 (A : B) (0–12.5 min). The flow rate was 1 mL min^−1^ and the injection volume was 5 μL.

GC‐MS analysis was performed using an Agilent 7000 E Triple Quadrupole GC/MS system with a J&W DB‐5 ms column (60 m×250 μm×0.25 μm). Column temperature 50 °C (initial) 10 °C min−1 to 240 °C (hold 1 min), injector temperature 280 °C, detector temperature 280 °C, helium carrier gas at 3 ml min−1, a source temperature of 200 °C and an ionization voltage of 70 eV. Spectra were collected by a ChemStation software package with a NIST mass spectral database. The mass range used was m/z 40–500.

## Conflict of Interests

The authors declare no conflict of interest.

1

## Supporting information

As a service to our authors and readers, this journal provides supporting information supplied by the authors. Such materials are peer reviewed and may be re‐organized for online delivery, but are not copy‐edited or typeset. Technical support issues arising from supporting information (other than missing files) should be addressed to the authors.

Supporting Information

## Data Availability

The data that support the findings of this study are available from the corresponding author upon reasonable request.
