# Supplementary material for: Exploring the Photocatalytic Cleavage Pathway of the β‐5 Linkage Lignin Model Compound on Carbon Nitride
Source: ChemSusChem. 2024 Nov 7;18(3):e202400955. doi: 10.1002/cssc.202400955 (PMC11789975; doi:10.1002/cssc.202400955)
Supplement: Supplementary file 1 — Supporting Information [file CSSC-18-e202400955-s001.pdf]

ChemSusChem

Supporting Information

Exploring the Photocatalytic Cleavage Pathway of the β-5 Linkage Lignin Model Compound on Carbon Nitride

Junhong Liu, Kathryn Ralphs, Christopher W. J. Murnaghan, Nathan Skillen, Gary N. Sheldrake, Philip McCarron, and Peter K. J. Robertson*

Supporting Information

EXPLORING THE PHOTOCATALYTIC CLEAVAGE PATHWAY OF THE β-5 LINKAGE LIGNIN MODEL COMPOUND ON CARBON NITRIDE.

Junhong Liu*[a], Kathryn Ralphs[a], Christopher W. J. Murnaghan[a], Nathan Skillen[a], Gary N. Sheldrake[a] Philip McCarron[b] and Peter K.J. Robertson*[a]

[a] School of Chemistry and Chemical Engineering, Queen's University Belfast, Belfast, BT9 5AG, U.K.

[b] IGFS, School of Biological Sciences, 19 Chlorine Gardens, Queen's University, Belfast, BT9 5DL, U.K.

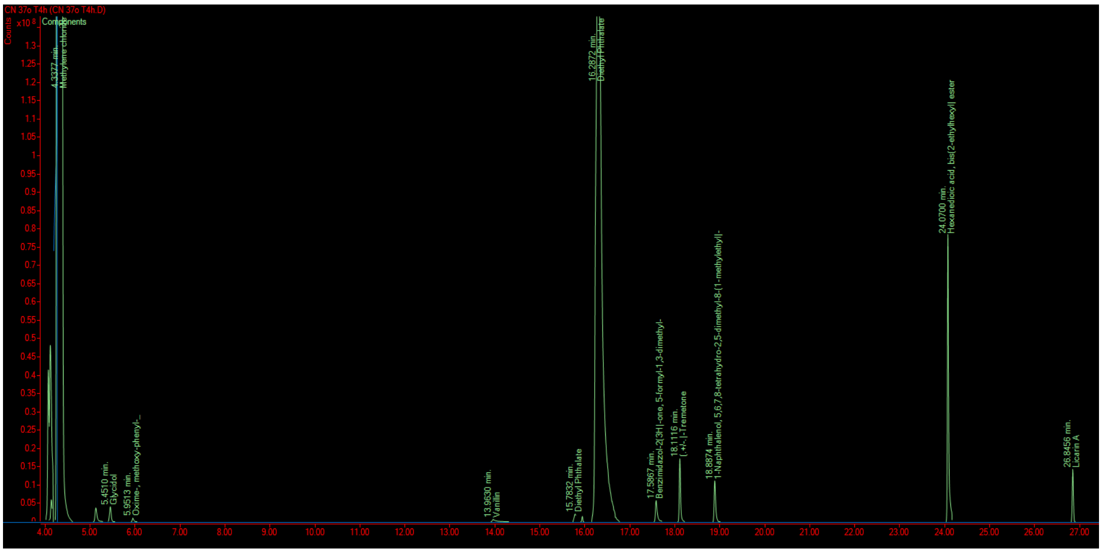

Fig.S1 GC-MS chromatography of substrate after 4 h reaction with CN.

Table S1 Peaks and corresponding m/z founded from 4h reaction substrate with CN under 370 nm illumination

Retention time (min)	Peak area (a.u.)	Peak m/z
4.07	81353718	44
4.12	159501291	44
4.14	9154870	43
4.26	828894319	42
4.34	980505579	84
5.13	6160858	56
5.45	4777517	44
5.95	839506	133
13.96	879036	152
15.78	1214872	149
15.94	560345	149
16.29	592620340	149
17.59	3390748	190
18.11	6419783	202
18.89	10130241	175
24.07	31081357	129
26.85	4254722	326

Table S2 Peaks and corresponding m/z founded from 24h reaction substrate with CN under 370 nm illumination

Retention time (min)	Peak area (a.u.)	Peak m/z
4.09	10825845	281
4.23	272817507	43
4.34	969212989	84
4.34	862978997	50
4.34	543748058	51
4.35	859713115	41
4.61	3792904	77
4.91	2671256	57
5.13	19547522	56
5.37	3840748	43
5.45	10909264	43
5.84	6963731	54
5.95	1104139	133
6.2	2244290	96
6.34	1010415	85
7.11	318305	281
7.23	1639353	53
9.84	17130922	69
15.94	616662	149
16.28	536587849	149
17.58	1795508	190
17.68	3295049	179
24.07	29996902	129

Table S3 Peaks and corresponding m/z founded from 24h reaction substrate with CN under 440 nm illumination

Component retention time (min)	Base Peak Area (a.u.)	Base Peak MZ
4.07	165568046	44
4.23	297005221	43
4.34	960065423	84
4.34	859549538	50
4.34	195288804	49
4.35	774062996	41
4.53	25982789	59
5.13	27404939	56
5.37	6834820	43
5.45	14421954	43
5.84	14248051	54
6.2	4818803	96
6.34	4974863	85
6.43	1598577	59
6.74	445165	110
7.23	2479258	53
9.05	2352642	69
9.6	1110895	95
9.83	35217005	69
11.12	1872243	123
11.57	937952	69
13.74	3594303	97
13.92	1660644	151
14.57	515240	164
14.69	661387	118
15.94	426899	149
16.28	423708363	149
17.57	20985059	190
17.66	15314369	179
18.11	1967310	202
18.37	1284134	206
18.58	5197730	178
18.88	4334180	175
24.07	28577715	129
24.42	1140516	177
26.84	3061135	326

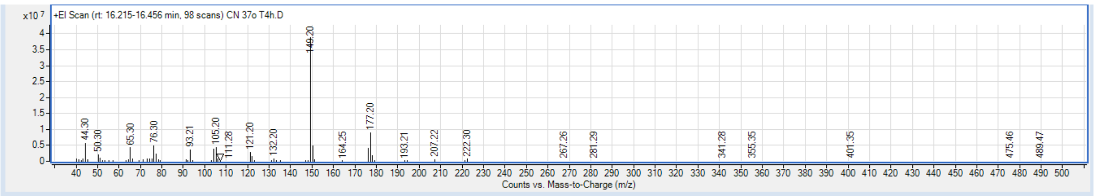

Fig.S2 Mass spectroscopy of Product P4

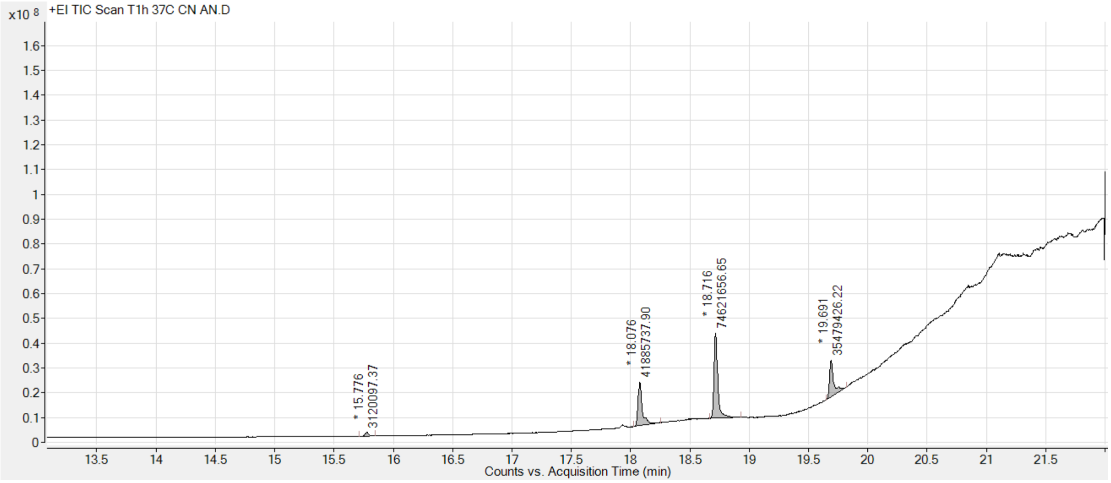

Fig.S3 GC-MS chromatography of substrate after 1 h reaction with CN.

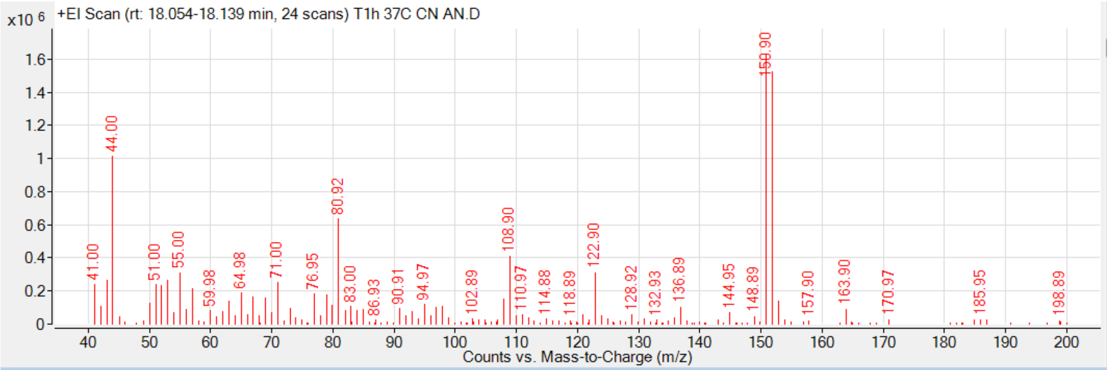

Fig.S4 Mass spectroscopy of Product P5

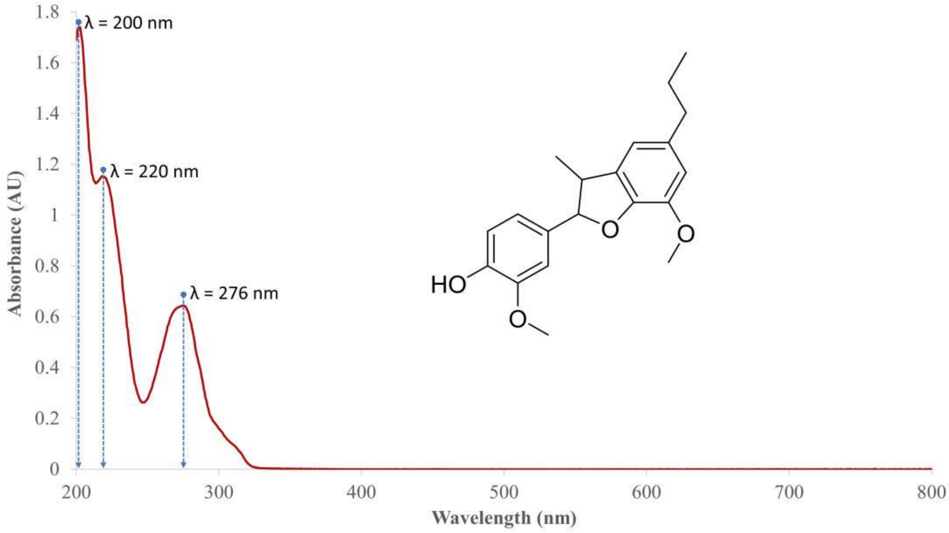

Fig.S5 UV-vis spectra for β-5 lignin model compound

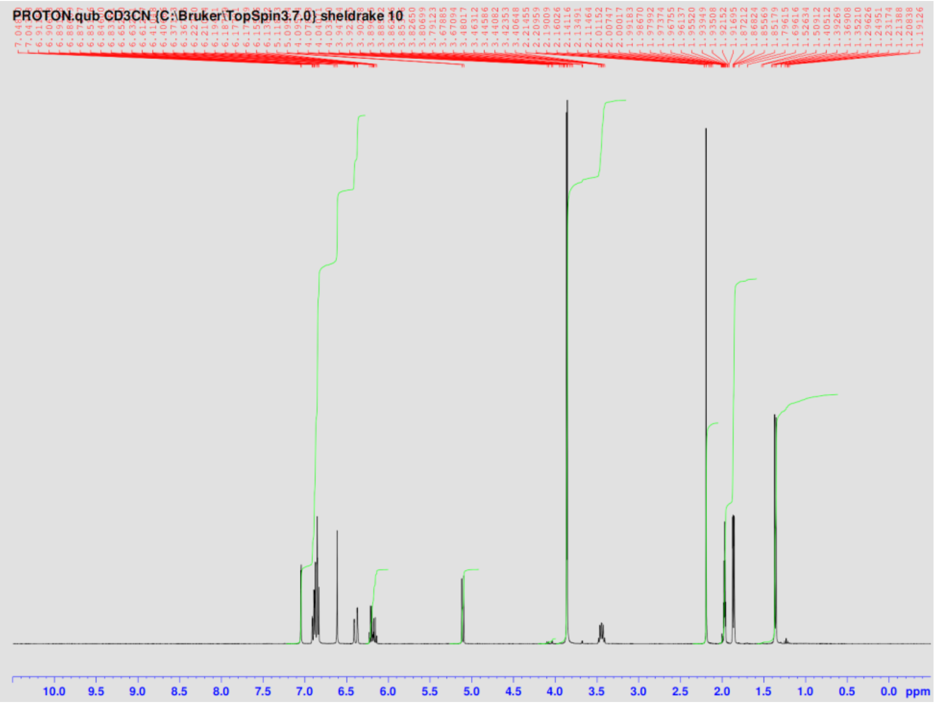

Fig.S6 NMR spectra of synthesized β-5 lignin model compound

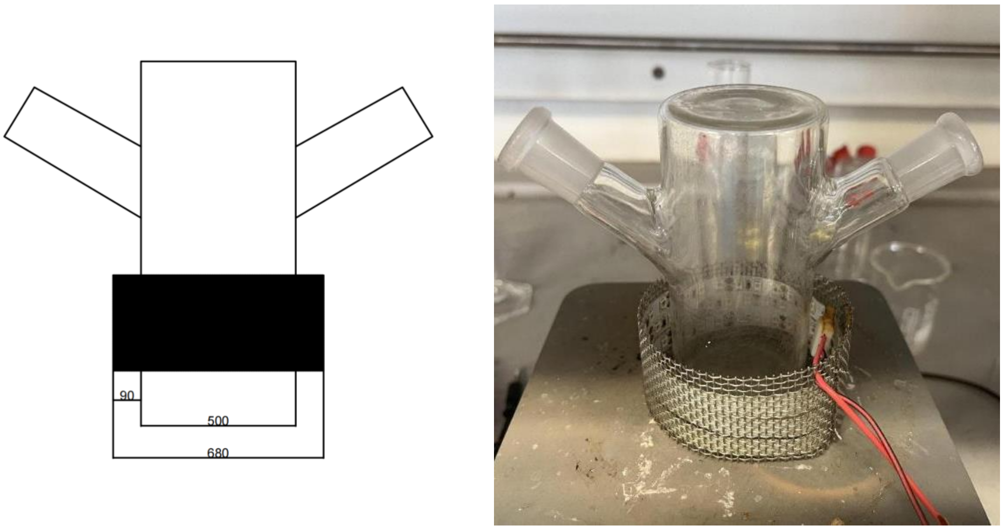

Fig. S7 Schematic and physical photographs of the reactor.

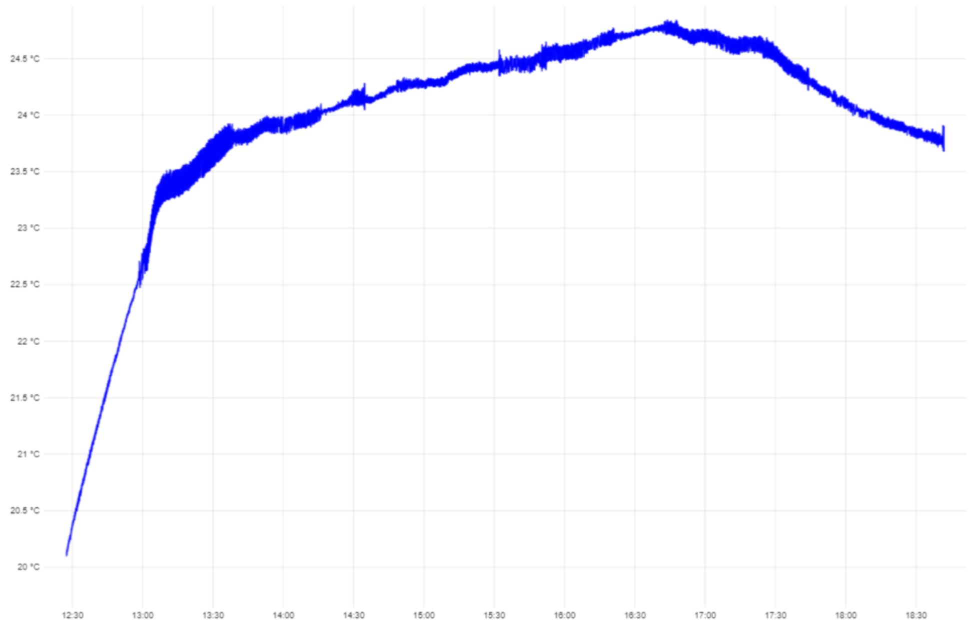

Fig. S8 Continuously recorded reaction temperature curve.

NMR Data

^{1}H NMR (400 MHz, $CDCl_3$) δ_H ppm 1.38 (3 H, d, J = 6.77 Hz, γ'-H), 1.87 (3 H, d, J = 6.53 Hz, γ''-H), 3.45(1H, m, β'-H), 3.88(3 H, s, OMe), 3.89(3 H, s, OMe), 5.10(1H, d, J = 9.45 Hz, α'-H), 5.62(1H, s, OH), 6.12(1H, m, J = 15.68 Hz, 6.59 Hz, Ar), 6.90(2 H, s, Ar), 6.97(1 H, s, Ar).

^{13}C NMR (400 MHz, $CDCl_3$) δ_H ppm 17.57, 18.40, 45.64, 55.94, 55.99, 93.92, 108.93, 109.23, 113.32, 114.08, 120.00, 123.52, 130.94, 132.1, 132.22, 133.28, 144.17, 145.79, 146.59, and 146.68
ES-HRMS (m/z)- [M+H+] calc mass 327.1596 mass found 327.1611
